# A One-Step RT-PCR-Coupled Cysteamine-Functionalized Gold Nanoparticle Assay for Colorimetric Detection of Tobacco Mosaic Virus

**DOI:** 10.3390/mps9040111

**Published:** 2026-07-27

**Authors:** Thuy-Duong Thi Tran, Quy Thi Vu, Hoa Thi Hoang, Phan Thi Ngoc Hoa, Nguyen Pham Thi Thao, Truong T. N. Lien

**Affiliations:** 1 Vietnam-Korea Institute of Science and Technology, Hoa Lac High-Teck Park, Hanoi 100000, Vietnam; thuyduongtranpharma@gmail.com (T.-D.T.T.); quy.nb2604@gmail.com (Q.T.V.);; 2 Center for Materials Innovation and Technology, VinUniversity, Hanoi 100000, Vietnam; 26nguyen.ptt@vinuni.edu.vn

**Keywords:** one-step RT-PCR, cysteamine-functionalized AuNPs, TMV, colorimetric assay

## Abstract

Viral diseases are one of the most destructive threats to global agriculture. Among plant viruses, tobacco mosaic virus (TMV) is a highly contagious pathogen infecting numerous economically vital crops. With no curable treatments, the only strategy to mitigate virus spread is early detection and plant removal. The gold standard for TMV detection is reverse transcriptase-polymerase chain reaction (RT-PCR), followed by agarose gel electrophoresis. To reduce TMV diagnosis time and eliminate the requirement for expensive equipment, this study developed and optimized a one-step RT-PCR-coupled cysteamine-functionalized gold nanoparticle assay for the colorimetric determination of the virus. By integrating cDNA synthesis and PCR amplification into a single tube and analyzing the results using cysteamine- functionalized gold nanoparticles (Au@Cys), the diagnosis turnaround time was significantly reduced. Furthermore, this AuNPs-based colorimetric assay enabled straightforward visual inspection with the naked eye, eliminating the need for costly optical devices. Additionally, our regression equation linking RT-PCR product color values, extracted as mean A value based on the CIELAB color space (green-red axis), and viral load allows for the accurate quantification of TMV infection levels in field samples. Our research lays the groundwork for the further development of more cost-effective, quantitative and rapid plant virus diagnosis methods.

## 1. Introduction

Viral diseases pose one of the greatest challenges in crop agriculture. To date, more than 2100 plant virus species have been identified, but most cause asymptomatic or mild diseases; therefore, they do not significantly affect crop yield and quality [1]. However, several viruses infecting important crops cause severe diseases, leading to massive agricultural losses of approximately $30 billion annually. Among plant viruses, tobacco mosaic virus (TMV), which belongs to the genus *Tobamovirus*, is well-characterized as it was the first virus discovered in history [2,3]. TMV has an exceptionally wide host range of over 885 plant species, including tobacco plants, eggplants, tomato, pepper and cucumber [4]. In Vietnam, TMV is one of the leading viruses threatening tobacco—a high-value crop—in the northern mountainous provinces [5]. Plants infected with TMV systematically develop severe symptoms such as light and dark green mottled patterns, chlorosis, necrosis and stunting in several parts of plants from leaves, stems to fruits, leading to significant losses of yield and quality [6,7,8].

TMV is shaped like a hollow cylinder, measuring 300 nm in length, 4 nm in inner diameter and 18 nm in outer diameter [9]. Its genetic material is a positive-sense single-stranded RNA (+ssRNA), which is protected by 2130 copies of the viral coat protein. These proteins are arranged along the longitudinal axis of the virus particle in a right-handed helix. The TMV genome encodes four proteins: an approximately 130 K protein that functions as an RNA helicase and methyltransferase; an approximately 180 K protein, generated via translational read-through of the 130 K protein termination codon, which contains an RNA-dependent RNA polymerase domain and forms a large subunit of the replicase complex; a 30 K protein involved in cell-to-cell movement; and a 17 K coat protein [10,11]. TMV is a highly stable and contagious virus. The major route for viral transmission among plants is through physical contact of wounded plant tissues with contaminated tools or surfaces [12,13].

Due to its ease of transmission, the primary strategy for disease control involves risk-reduction measures such as identifying and removing infected plants [1]. To date, the standard method for accurate TMV detection has been reverse transcriptase-polymerase chain reaction (RT-PCR) [14]. In this method, total RNA extracted from leaf samples is used to synthesize cDNA, which then serves as a template for PCR with a TMV-specific primer pair. The PCR results are analyzed using agarose gel electrophoresis. To reduce the experimental time and minimize the need for expensive equipment, we optimized a one-step RT-PCR procedure coupled with a gold nanoparticle-based colorimetric assay for visual TMV detection. Both cDNA synthesis and PCR are conducted in a single reaction, and the RT-PCR product is subsequently analyzed by naked eye using gold nanoparticles (AuNPs). Characterized by size, shape and aggregation-dependent optical properties, AuNPs serve as an excellent foundation for colorimetric biosensors [15]. Our procedure holds potential for the further development of user-friendly point-of-care tests for rapid viral detection in plants by farmers.

## 2. Materials and Methods

### 2.1. Materials and Chemical Reagents

All chemicals and biological reagents employed in this study were of analytical grade and were utilized without additional purification. Chloroauric acid tetrahydrate (HAuCl_4_·4H_2_O) and trisodium citrate (Na_3_C_6_H_5_O_7_), sodium chloride (NaCl) were obtained from Sigma-Aldrich (Saint Louis, MO, USA). Molecular biology reagents, including SuperScript™ IV One-Step RT-PCR System 100 reactions (12594100), GeneRuler 100 bp DNA Ladder (M100), TriTrack DNA Loading Dye (6×) (R1161), Top Vision Agarose (R0491), and 10× TBE buffer (B52), were purchased from Thermo Scientific (Waltham, MA, USA). RedSafe nucleic acid staining reagent (21141) was purchased from iNtRON Biotechnology (Seongnam-si, Republic of Korea). Cysteamine (Cys) was obtained from Merck (Darmstadt, Germany). All primers were synthesized and purified by Integrated DNA Technologies (IDT, Coralville, IA, USA).

### 2.2. Synthesis and Surface Functionalization of Gold Nanoparticles (AuNPs)

AuNPs with diameters of 15–17 nm were synthesized via the citrate reduction method (Turkevich–Frens), as described in our previous work [16]. The synthesized AuNPs were collected by centrifugation at 10,000 rpm for 30 min and redispersed in deionized water to obtain a concentrated suspension with an optical density (OD) of 20. This AuNP mixture storage form was designed for long-term stability. Before use, the stock AuNP solution was thoroughly vortexed to ensure uniform dispersion and then diluted with deionized water to achieve the desired concentration. The cysteamine-coated AuNP (Au@Cys) solution was prepared by mixing the diluted AuNP with 0.01 mM cysteamine and incubating the mixture at room temperature for at least one hour.

The physicochemical properties of the AuNPs and Au@Cys were comprehensively characterized. UV–Vis absorption spectra were recorded using a V-770 spectrophotometer (JASCO, Tokyo, Japan). The hydrodynamic diameter and surface charge were determined via dynamic light scattering using a Zetasizer Nano ZS (Anton Paar, Graz, Austria). Furthermore, the morphology and particle size distribution were visualized using transmission electron microscopy (TEM) at 200 kV (JEM-2011F, JEOL, Tokyo, Japan).

### 2.3. Plant Material and Total RNA Extraction

Tobacco (*Nicotiana tabacum*) and tomato (*Solanum lycopersicum*) leaf samples were collected from cultivation areas in Cao Bang province, Viet Nam. Samples were stored under appropriate conditions prior to analysis.

Total RNA was extracted using the RNeasy Plant Mini Kit (74904, Qiagen, Hilden, Germany) according to the manufacturer’s protocol. Briefly, 0.1 g of leaf tissue was ground to a fine powder in liquid nitrogen using a pre-chilled mortar and pestle. The homogenate was transferred to a 2 mL microcentrifuge tube, lysed with 450 µL of RLT buffer (DTT added), and loaded onto a QIAshredder spin column. After centrifugation at 13,400 rpm for 2 min, the supernatant (~400 µL) was recovered and mixed with 200 µL of absolute ethanol (96–100%). The mixture was transferred to an RNeasy spin column and centrifuged at 13,400 rpm for 15 s. The column was washed sequentially with 700 µL of RW1 buffer and twice with 500 µL of RPE buffer. RNA was eluted in 50 µL of RNase-free water and stored at −80 °C.

### 2.4. Reverse Transcription—Polymerase Chain Reaction (RT-PCR)

In this study, specific primers targeting the TMV coat protein (CP) gene (FP: 5′-TCTTGTCATCAGCGTGGGC-3′; RP: 5′-CCAGAGGTCCAAACCAAACCA-3′) were used to yield a 430 bp product [17]. To enable subsequent interaction with the gold nanoparticles, these primers were chemically synthesized with a 5′ thiol modification (5′ Thiol Modifier C6 S-S) by Integrated DNA Technologies (IDT).

One-step RT-PCR was performed using the SuperScript™ IV One-Step RT-PCR System (K1622, Thermo Scientific). A 10 µL reaction was prepared by mixing 5 µL 2× Master Mix, 200 ng of total RNA, 0.2 µM of forward and reverse primers, 0.1 µL RT mix, and adjusted to 10 µL with nuclease-free water. The thermal profile consisted of reverse transcription (RT) at 50 °C for 10 min, followed by 98 °C for 2 min to stop the RT step; 30 cycles of 98 °C for 10 s, 58 °C for 10 s, and 72 °C for 15 s; and a final extension at 72 °C for 5 min. Amplicons were analyzed by 2% agarose gel electrophoresis in 1× TBE buffer at 100 V and visualized against a 100 bp DNA ladder (Thermo Scientific).

### 2.5. Recombinant Plasmid Standard Preparation

The target sequence was amplified using DreamTaq RT-PCR Master Mix with a 15 min final extension to facilitate 3′-A tailing. Amplicons were cloned into the pCR 2.1-TOPO TA vector (Invitrogen, Carlsbad, CA, USA) and transformed into chemically competent *E. coli* TOP10 cells. Positive clones were identified via blue-white screening on LB agar containing 50 µg/mL kanamycin and X-gal. The inserts were confirmed by colony RT-PCR and Sanger sequencing (Apical Scientific, Selangor, Malaysia). Recombinant plasmids were extracted using the GeneJET Plasmid Miniprep Kit (Thermo Scientific) and quantified via NanoDrop Eight Spectrophotometry. Plasmid copy number per microliter was calculated according to the following equation:

$$C=\frac{\left([DNA]\times 6.022\times 10^{23}\right)}{P\times 660}$$

where C is the plasmid copy number per microliter, [DNA] represents the DNA concentration (g × µL^−1^), P is the total plasmid length in base pairs (bp), 660 g × mol^−1^ is the average molecular weight of a double-stranded DNA base pair, and 6.022 × 10^23^ molecules × mol^−1^ is Avogadro’s constant. The total plasmid length was determined by the sum of the pCR 2.1-TOPO TA vector (3931 bp) and the TMV insert (430 bp). Then, a 10-fold serial dilution (10^8^ to 10^1^ copies/µL) was prepared in nuclease-free water, and one microliter of each dilution served as the RT-PCR template for further experiments.

## 3. Results

### 3.1. Evaluation of Cysteamine-Functionalized AuNPs for High-Contrast Colorimetric Sensing

The characteristic ruby-red color of monodisperse, citrate-capped gold nanoparticles (AuNPs) arises from localized surface plasmon resonance (LSPR) [18]. Because the colloidal stability of citrate-stabilized AuNPs is highly sensitive to ionic environments, the addition of electrolytes like NaCl screens the surface charge, lowering interparticle repulsion and allowing attractive van der Waals forces to drive aggregation. This aggregation shifts the plasmonic coupling between neighboring AuNPs, causing a bathochromic LSPR shift and a distinct visual transition from red to purple or blue. To exploit this mechanism in colorimetric biosensing systems, establishing an efficient surface protective layer to modulate salt-induced aggregation behavior becomes a critical requirement. To achieve this, cysteamine (2-aminoethanethiol), a small molecule containing a thiol group (-SH) and an amine group (-NH_2_), was employed for surface functionalization. The thiol group binds covalently to the AuNP surface through strong gold-sulfur (Au-S) interactions, while the exposed, positively charged amine groups facilitate the binding efficiency of the negatively charged dsDNA amplicons.

**Figure 1:**
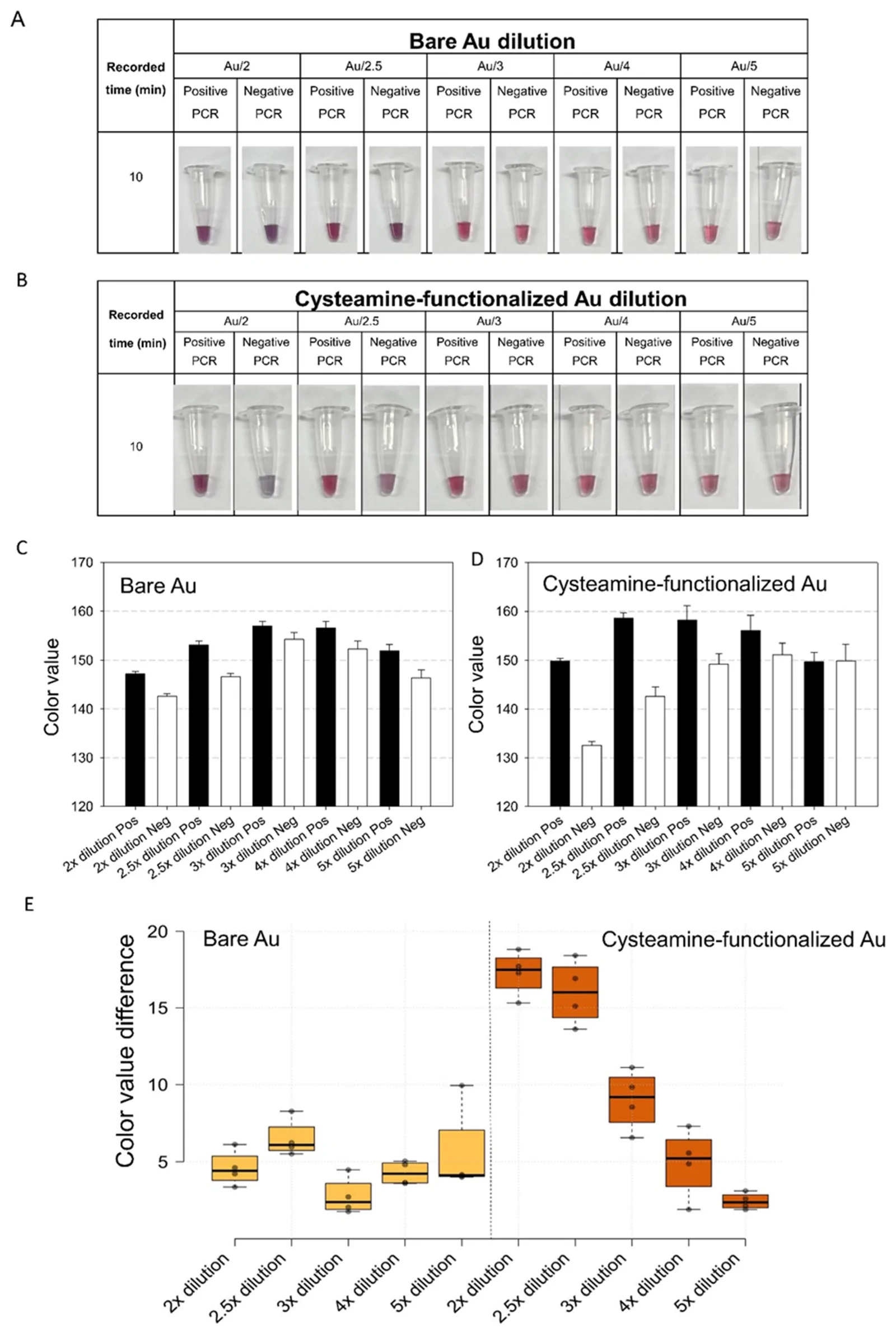
Cysteamine-functionalized gold nanoparticles enhance colorimetric discrimination. (**A**) Color changes in citrate-capped (bare) gold nanoparticles in the presence of positive RT-PCR amplicons and negative controls. (**B**) Color changes in cysteamine-functionalized gold nanoparticles in the presence of positive RT-PCR amplicons and negative controls. Both bare and cysteamine-functionalized gold nanoparticles were prepared by diluting concentrated gold nanoparticles (OD_520_ = 20) to various dilution factors. Then, 10 µL of bare or cysteamine-functionalized gold nanoparticles was incubated with 1 µL of RT-PCR products (diluted in 9 µL distilled water) at 37 °C for 15 min, followed by the addition of 2 µL of 3 M NaCl. Visual color changes were captured 10 min after salt addition. (**C**) Color value of the samples in (**A**), extracted as the mean A value based on the CIELAB color space (green-red axis). (**D**) Color value of the samples in (**B**), extracted as the mean A value based on the CIELAB color space (green-red axis). Data are presented as mean ± s.e.m (*n* = 4). At least two independent experiments were performed with similar results. (**E**) Color value difference (Δ color value) between positive and negative samples calculated from (**C**,**D**).

To optimize the visual discrimination between positive and negative RT-PCR samples, the colorimetric response of unmodified AuNPs and Cysteamine-modified AuNPs (Au@Cys) was evaluated at a range of dilution factors (2×, 2.5×, 3×, 4×, and 5×). The positive sample used in this study was a plasmid containing a DNA sequence encoding the TMV coat protein (10^8^ copies/µL), while distilled water served as the negative control. To optimize a diagnostic procedure for TMV—an RNA virus—one-step RT-PCR reactions were performed to amplify the TMV-specific sequence (430 bp) in both samples (Figure S2). The resulting products were subsequently subjected to a colorimetric assay. First, the stock AuNP solution (OD_520_ = 20) was serially diluted and incubated with negative and positive RT-PCR samples. The colorimetric responses were recorded 10 min post salt induction. As illustrated in Figure 1A, visual discrimination between positive and negative RT-PCR samples incubated with unmodified AuNPs is highly challenging at dilution factors of 3× or greater, as both positive and negative samples retained a similar red/pink hue. Although lower dilutions (2× and 2.5×) enhanced naked-eye contrast, quantitative assessment of color value showed a narrow difference (2.8–6.5 units) across all groups (Figure 1C,E). This narrow colorimetric difference limits distinct positive/negative classification and poses a challenge for the limit of detection (LOD) of the biosensing system, especially for samples with low viral titer. However, surface functionalization with cysteamine markedly improves the colorimetric shift. The system shows the clearest color discrimination at the dilution factors of 2× and 2.5× (Figure 1B,D). At these dilutions, the positive samples maintained their dispersed red/pink state, which can be attributed to the electrostatic stabilization mediated by the interaction between the negatively charged phosphodiester backbone of the dsDNA amplicons and the amino groups of Au@Cys. Conversely, the negative controls underwent rapid salt-induced aggregation, transitioning into a distinct purple-gray hue. The boxplots quantifying the color value difference highlight the advantage of the surface modification, where the color value gaps between the positive control and the negative control increase significantly (Figure 1E). Although the 2× dilution exhibited the highest color value difference, the 2.5× dilution was preferred as it yielded a brighter red signal for positive samples (Figure 1B,D).

**Figure 2:**
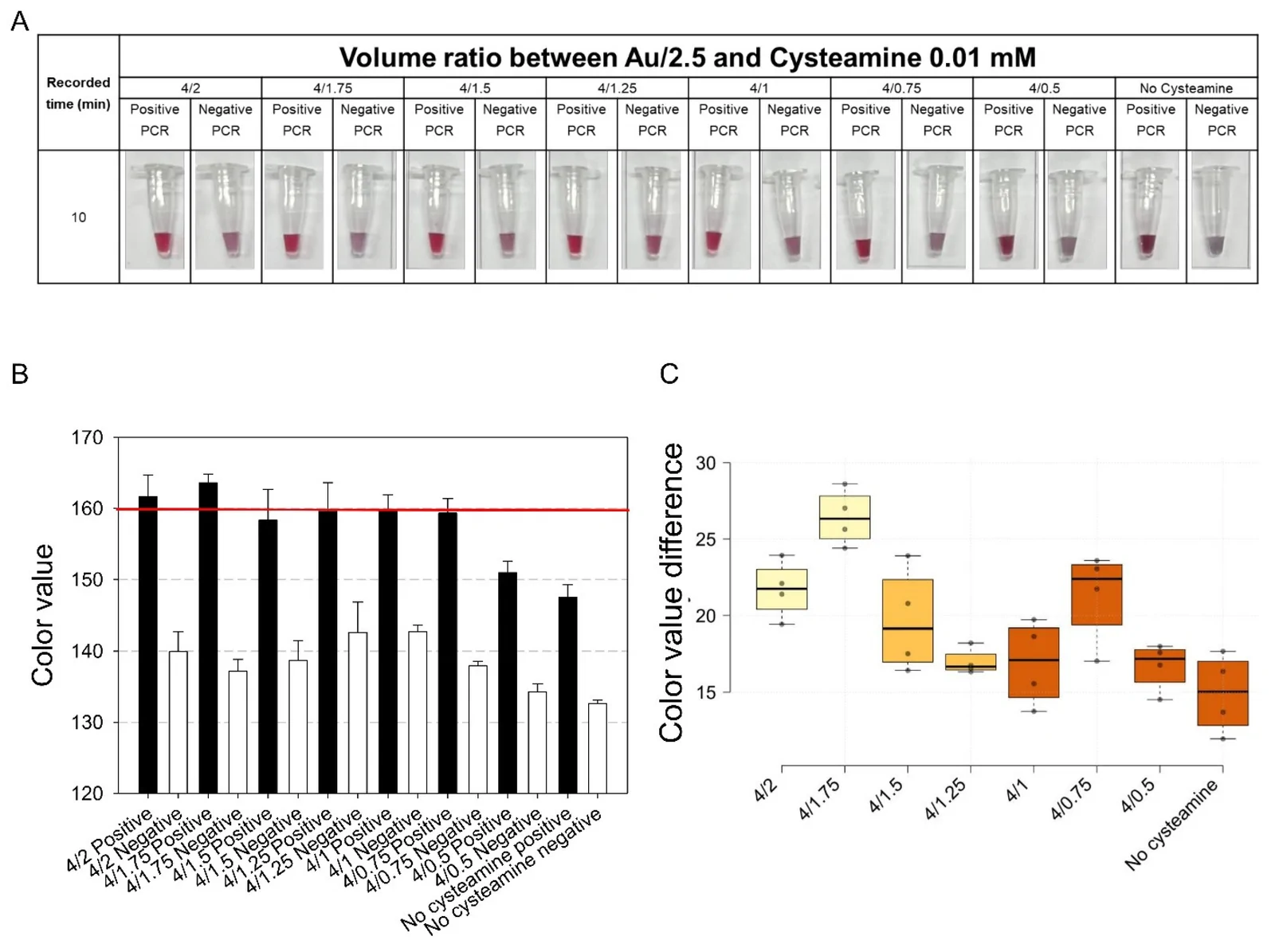
Effect of cysteamine concentration on colorimetric discrimination. (**A**) Color changes in cysteamine-functionalized gold nanoparticles in the presence of positive RT-PCR amplicons and negative controls evaluated at various volume ratios of gold nanoparticles to 0.01 mM cysteamine. (**B**) Color value of the samples in (**A**), extracted as the mean A value based on the CIELAB color space (green-red axis). The red line represents the threshold for a stable colloidal state. Data are presented as mean ± s.e.m (*n* = 4). At least two independent experiments were performed with similar results. (**C**) Color value difference (Δ color value) between positive and negative samples calculated from (**B**).

Next, we evaluated the volume ratio between the 2.5× diluted AuNP solution and 0.01 mM cysteamine to maximize positive-to-negative color discrimination while maintaining standard brightness for positive samples. Colorimetric changes were captured 10 min after NaCl addition (Figure 2A), and the color value graph was shown in Figure 2B, using a red baseline (y = 160) to mark the minimum value required for brightness in positive controls. Figure 2A reveals that the Au and cysteamine ratio affects the colloid state and colorimetric shift when interacting with RT-PCR targets. At low cysteamine amount (4/0.5), inadequate surface coverage prevents the formation of sufficient electrostatic bridges with amplicons. Consequently, even positive samples suffer severe salt-induced aggregation, turning dark and shifting from red to purple-gray (Figure 2A). Quantitative data confirmed this, showing color values far below 160 units (Figure 2B). Conversely, higher cysteamine ratios (from 4/0.75 to 4/2) preserved a vibrant, clear red/pink hue in positive samples, allowing facile positive/negative discrimination by the naked eye (Figure 2A,B). The 4/1.75 ratio yielded the maximum color value difference between the negative and positive controls (Figure 2C); therefore, this specific ratio is selected for subsequent optimization steps.

Transmission electron microscopy (TEM), UV-Vis spectroscopy, and zeta potential were employed to further elucidate the structural integrity and surface modification of the nanoparticles in each functionalization stage. As illustrated in Figure S1A,B, both the bare AuNPs and the cysteamine-modified AuNPs (Au@Cys) exhibit highly uniform, monodisperse spherical morphologies. The absence of clustering in Figure S1B indicates that the cysteamine does not affect the colloidal stability or induce premature aggregation. This is further confirmed by UV-VIS spectroscopy results (Figure S1D). The absorption peaks of the gold particles before and after functionalization with cysteamine remained unchanged. To confirm that the nanoparticles are functionalized with cysteamine, the zeta potential was measured, and the results are shown in Figure S1E. Zeta potential measurements showed that Au@Cys are more positive than bare AuNPs (about −38 mV compared to −46 mV), proving the effect of cysteamine on the surface of citrate-capped gold nanoparticles. Crucially, the introduction of positive amine groups NH3^+^ onto the AuNPs serves as an electrostatic bridge that elevates the binding efficiency of the negatively charged phosphodiester backbone of dsDNA amplicons. Following dsDNA incubation (Figure S1C), the Au@Cys remained exceptionally well-dispersed, exhibiting an increased inter-particle distance. This expanded separation is driven by a robust DNA corona in AuNPs, creating a dense layer of steric hindrance and enhanced long-range electrostatic repulsion. As a result, this structure effectively protects nanoparticles against NaCl-induced aggregation. In Figure S1D, upon salt induction, the LSPR peak of the bare citrate-capped AuNPs shifts significantly to 534 nm, accompanied by a broader absorbance tail at longer wavelengths, indicating partial nanoparticle aggregation under high ionic strength. In contrast, the LSPR peak of Au@Cys shows only a minor shift to 528 nm with substantially less spectral broadening, suggesting that cysteamine surface modification facilitates the formation of a more robust DNA corona on the AuNP surface, thereby enhancing resistance to salt-induced aggregation.

### 3.2. Role of Thiol-Functionalized Primers in the Au@Cys Colorimetric Assay

**Figure 3:**
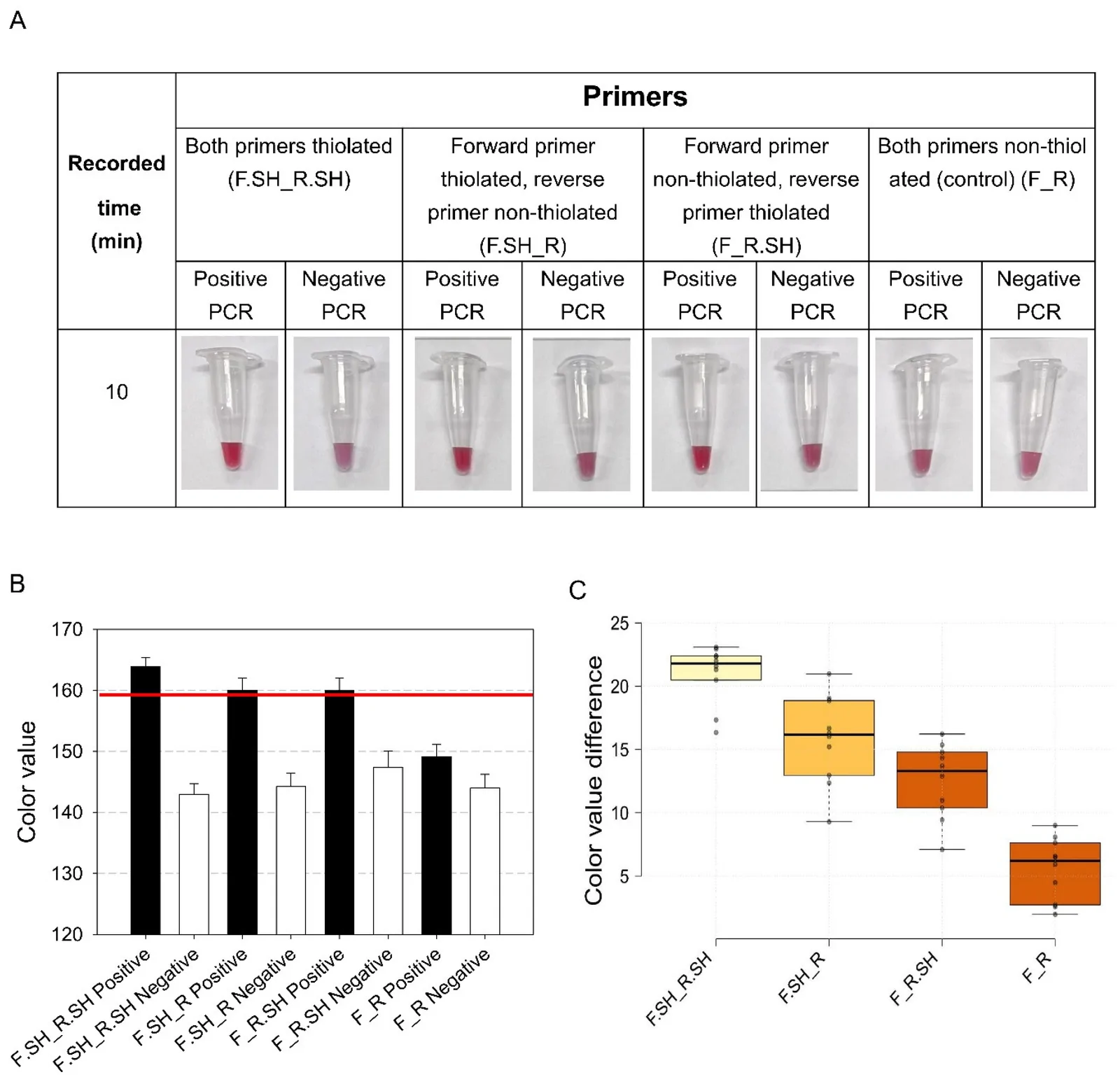
Effect of primer thiolation on colorimetric discrimination. (**A**) Color changes in cysteamine-functionalized gold nanoparticles in the presence of positive RT-PCR amplicons and negative controls. RT-PCR amplifications were performed using four primer combinations: dual-thiolated (F.SH_R.SH), single-thiolated (F.SH_R or F_R.SH), and non-thiolated (F_R) primers. (**B**) Color value of the samples in (**A**), extracted as the mean A value based on the CIELAB color space (green-red axis). The red line represents the threshold for a stable colloidal state. Data are presented as mean ± s.e.m (*n* = 10). Five independent RT-PCR experiments utilizing two independent thiolated-primer batches were performed with similar results. (**C**) Color value difference (Δ color value) between positive and negative samples calculated from (**B**).

To investigate the role of thiolated primers in the interactions between RT-PCR amplicons and Au@Cys, four pairs of primers, including both primers thiolated, either the forward or reverse primer thiolated and both primers non-thiolated, were used to conduct RT-PCR. The results presented in Figure 3 demonstrate that thiol-functionalized primers are critical for anchoring DNA onto Au@Cys, directly affecting the colorimetric response and colloidal stability.

With dual thiolated primers (F.SH_R.SH) or single thiolated variants (F.SH_R or F_R.SH), the positive RT-PCR control retained a bright red color, whereas the negative RT-PCR control gradually shifted to purple (Figure 3A). The contrast in color value between positive and negative samples gradually diminishes as a function of primer thiolation levels (Figure 3B,C). This confirms that thiolated dsDNA amplicons effectively stabilize Au@Cys against electrolyte-induced aggregation, preserving their characteristic LSPR state. This enhanced stability stems from the strong affinity of thiol groups to the gold surface via Au–S bonding. The immobilized dsDNA provides sufficient steric hindrance and long-range electrostatic repulsion to prevent aggregation under high salt concentrations. In contrast, the non-thiolated group (F_R) showed similar colloidal stability between positive and negative controls, leading to difficulties in distinguishing colors (Figure 3A,C). Without thiol groups, dsDNA cannot covalently anchor to the gold surface, relying instead on weak electrostatic attraction or non-specific physical adsorption. These interactions fail to resist salt-induced charge screening, triggering nanoparticle aggregation. This confirms that the presence of non-thiolated dsDNA cannot stabilize Au@Cys matrices and thiol-mediated anchoring is required for stable bonding. In conclusion, primer thiolation is the crucial factor for Au@Cyscolorimetric assays, with dual-thiolated systems offering the highest stabilization and optimal positive-to-negative visual contrast (Figure 3B,C).

### 3.3. Optimization of RT-PCR Product Dilution to Minimize Matrix Interference

**Figure 4:**
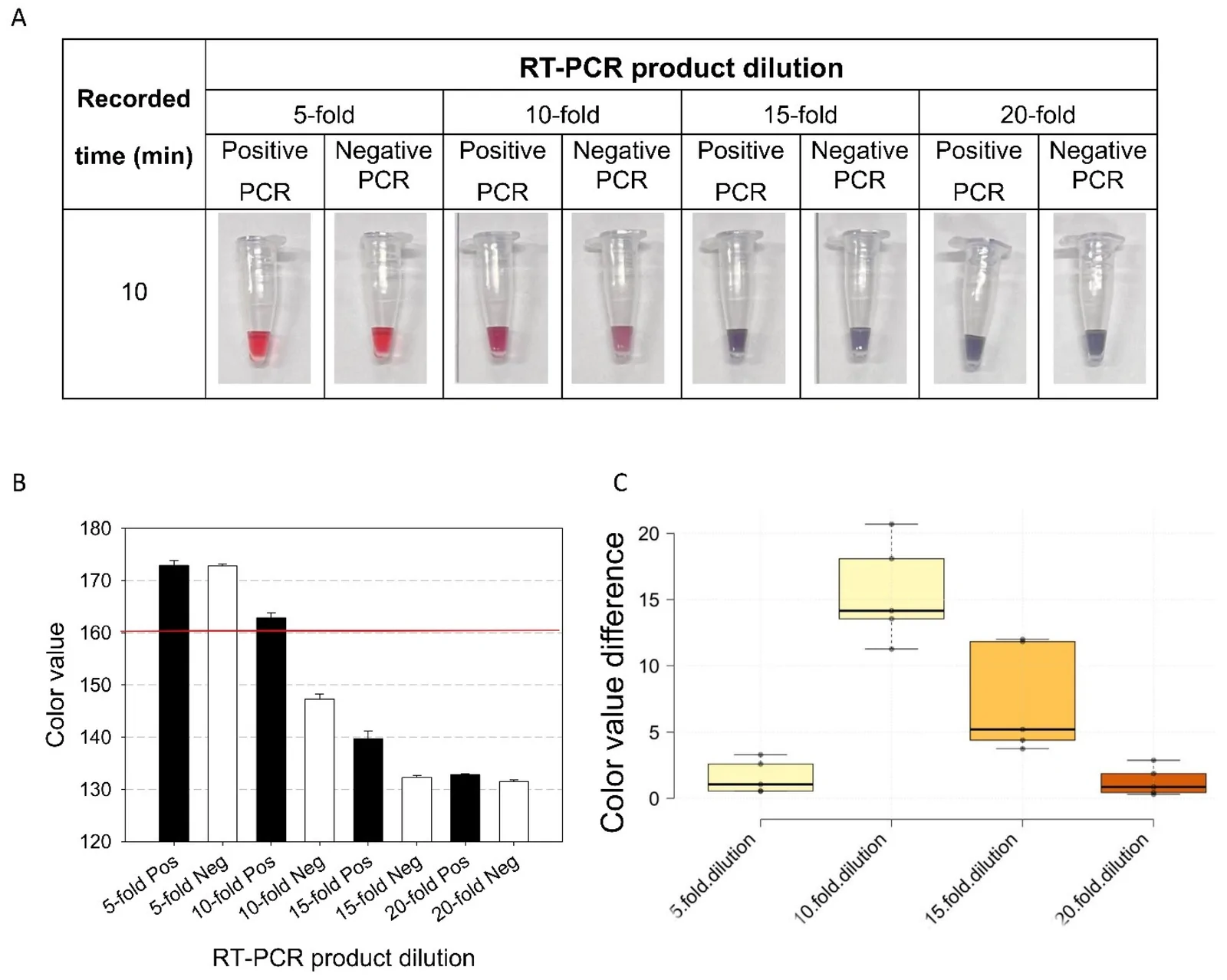
Effect of RT-PCR amplicon dilution on colorimetric discrimination. (**A**) Color changes in cysteamine-functionalized gold nanoparticles in the presence of positive RT-PCR amplicons and negative controls, evaluated at 5-, 10-, 15-, and 20-fold dilutions. (**B**) Color value of the samples in (**A**), extracted as the mean A value based on the CIELAB color space (green-red axis). The red line represents the threshold for a stable colloidal state. Data are presented as mean ± s.e.m (*n* = 5). At least two independent experiments were performed with similar results. (**C**) Color value difference (Δ color value) between positive and negative samples calculated from (**B**).

The optimization of the RT-PCR product dilution factor is fundamental to establishing a highly discriminative and reliable colorimetric assay. This parameter minimizes the complex effect of the RT-PCR components (Mg^2+^ ions, dNTPs, and residual enzymes) on the nanoparticle surface chemistry. Furthermore, finding the optimal dilution factor is essential to establish a kinetic equilibrium and avoid false positives in negative controls due to insufficient dilution or false negatives in positive controls due to overdilution. As shown in Figure 4, at a low dilution (5-fold), both positive and negative samples exhibit a similar red coloration (Figure 4A) with almost equal values (~172 units) (Figure 4B), yielding a contrast delta near zero (Figure 4C). This indicates that excessive residual primers non-specifically shield the AuNP surface and prevent salt-induced aggregation even in the negative sample, causing false positive results. Conversely, at high dilutions (15-fold and 20-fold), both positive and negative samples rapidly fade below the red baseline, turning into a purple-blue hue (between 132 and 140 units) (Figure 4A,B). The collapse of the colloidal system in the positive sample indicates insufficient amplicon density to prevent salt-induced aggregation. A 10-time dilution of the RT-PCR amplicons proved optimal for positive and negative controls through surface plasmon resonance shifts. At this dilution level, target DNA sequences in positive samples exert adequate electrostatic and steric stabilization, protecting cysteamine-functionalized AuNPs against salt-induced aggregation and retaining the red color under high-electrolyte conditions (Figure 4A,B). In contrast, negative samples undergo significant fading, with the highest color value difference between positive and negative samples (Figure 4C), confirming that the lack of target DNA allows surface charge neutralization and immediate colloidal destabilization.

### 3.4. Effect of Incubation Temperature and Duration on Colorimetric Response

**Figure 5:**
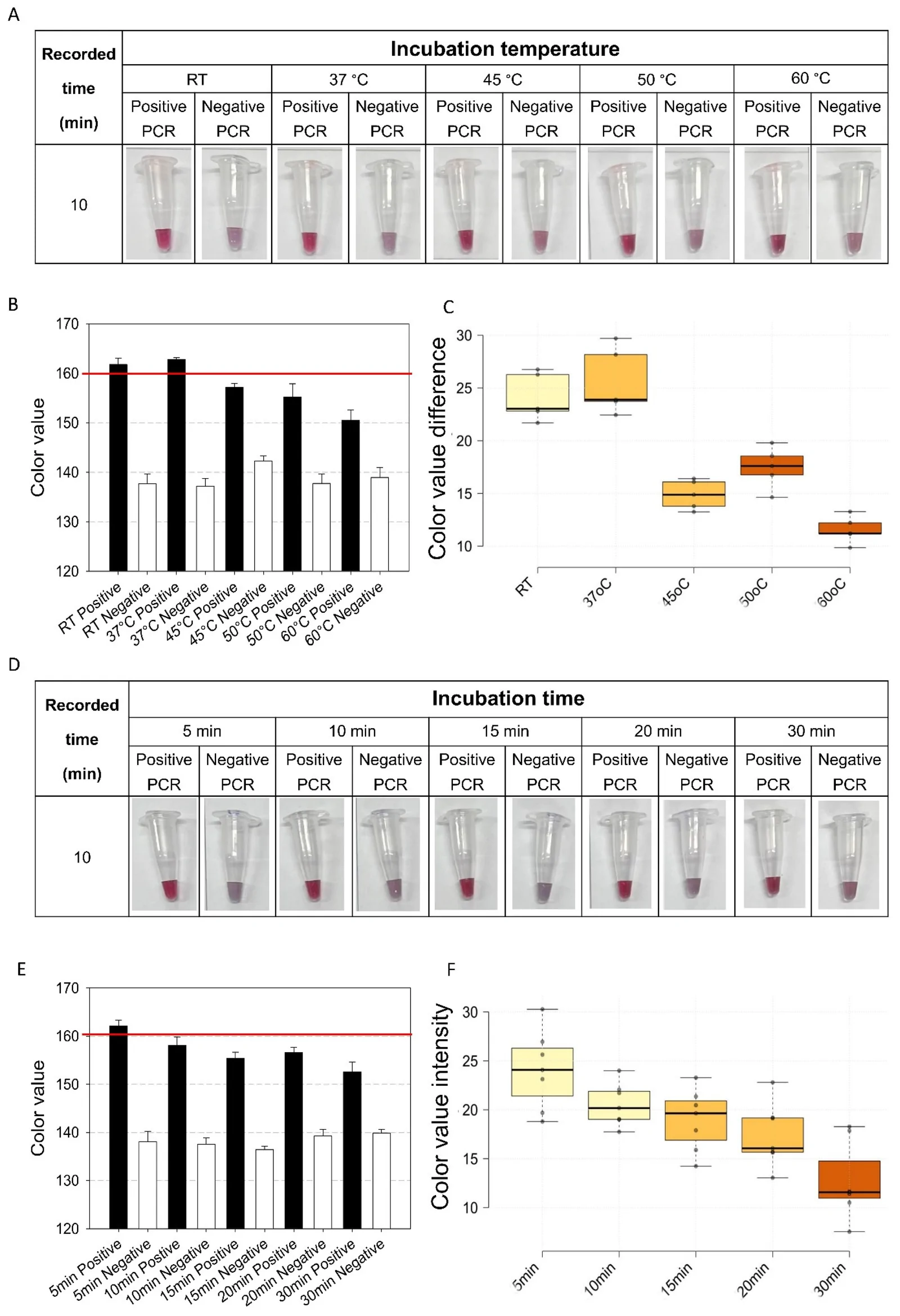
Effect of incubation temperature and duration on colorimetric discrimination. (**A**) Color changes in cysteamine-functionalized gold nanoparticles in the presence of positive RT-PCR amplicons and negative controls, evaluated at various incubation temperatures for 15 min prior to salt addition. (**B**) Color value of the samples in (**A**), extracted as the mean A value based on the CIELAB color space (green-red axis). The red line represents the threshold for a stable colloidal state. Data are presented as mean ± s.e.m (*n* = 5). At least two independent experiments were performed with similar results. (**C**) Color value difference (Δ color value) between positive and negative samples calculated from (**B**). (**D**) Color changes in cysteamine-functionalized gold nanoparticles in the presence of positive RT-PCR amplicons and negative controls, evaluated across various incubation durations. (**E**) Color value of the samples in (**D**), extracted as the mean A value based on the CIELAB color space (green-red axis). The red line represents the threshold for a stable colloidal state. Data are presented as mean ± s.e.m (*n* = 7). At least two independent experiments were performed with similar results. (**F**) Color value difference (Δ color value) between positive and negative samples calculated from (**E**).

To ensure maximum assay reproducibility and a high-contrast colorimetric assay, the kinetic parameters governing the incubation between the RT-PCR amplicons and the Au@Cys nanoparticles, specifically temperature and duration, were systematically optimized. Controlling the incubation temperature is vital to promote effective collision frequencies between the nanomaterials and the DNA, facilitating both the initial electrostatic binding and the subsequent Au-S chemisorption. Concurrently, optimizing the incubation duration is required to achieve complete interfacial saturation on the nanoparticle while preventing premature colloidal destabilization potentially triggered by low-concentration electrolytes present within the RT-PCR dilution. As shown in Figure 5, the best color value difference between positive and negative samples is achieved at mild temperatures (room temperature and 37 °C) with a short incubation time of only 5 min (Figure 5A,C,D,F). Mechanistically, this fast interaction happens because the positive amine groups (NH_3_^+^) on the Au@Cys nanoparticles strongly attract the negatively charged phosphate backbone of the DNA. This DNA barrier protects the AuNPs in positive samples from salt-induced aggregation, keeping the solution bright red and color value above the threshold line (y = 160) (Figure 5A,B,D,E). In contrast, high temperatures (45 °C to 60 °C) may cause thermal fluctuations, whereas extended incubation times up to 30 min allow residual salts from the RT-PCR buffer to trigger premature, non-specific colloidal destabilization. Overall, a brief 5 min incubation at room temperature is the ideal condition. It provides a fast, simple, instrument-free, and reliable test that is perfectly suited for point-of-care diagnostic applications.

### 3.5. Sensitivity, Specificity, and Field Sample Validation of the Au@Cys Nanoparticles Assay

To evaluate the qualitative and quantitative analytical performance of the Au@Cys-based colorimetric biosensing system, a 10-fold serial dilution of the plasmid containing the target TMV coat protein gene sequence was prepared. The template amount ranged from 10^8^ to 10^1^ copies/reaction, with distilled water serving as the negative control (Neg). Before colorimetric detection, the amplification performance of RT-PCR was also verified using agarose gel electrophoresis with RT-PCR products of a dilution series of plasmid templates (Figure S2). Distinct and intense bands were observed from 10^8^ to 10^3^ copies of template/reaction, while no visible bands appeared at 10^2^ copies of template/reaction and lower, suggesting that the RT-PCR with agarose gel detection threshold was at 10^3^ copies of template/reaction under current conditions. These amplicons were subsequently used in the Au@Cys colorimetric assay to validate sensitivity. The sensitivity was assessed through both visual inspection and color value analysis to determine the limit of detection (LOD).

**Figure 6:**
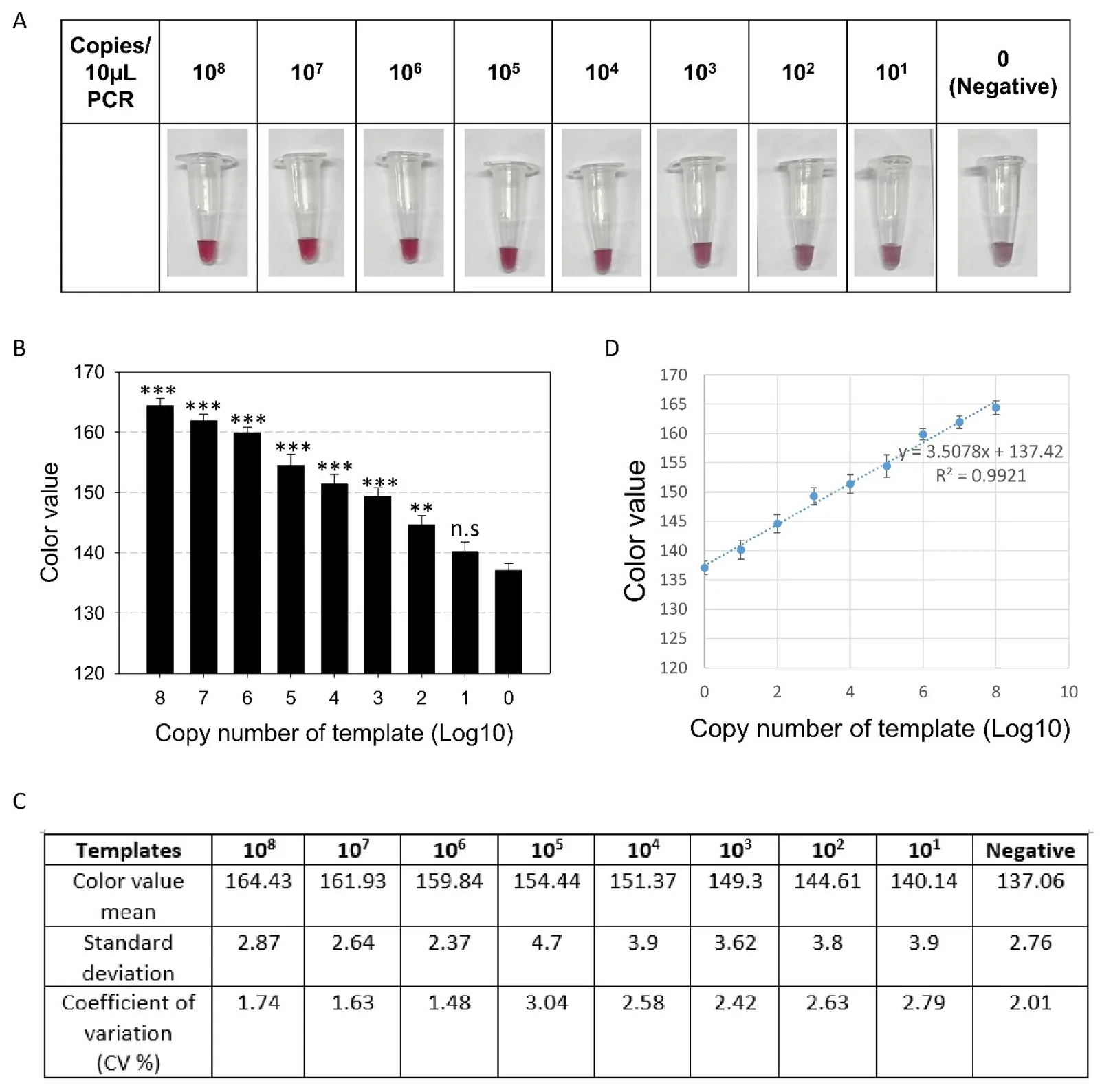
Analytical sensitivity and linearity of the colorimetric assay using standard samples. Standard DNA samples with known copy numbers of the gene encoding the tobacco mosaic virus (TMV) coat protein were used as templates for one-step RT-PCR. The resulting RT-PCR amplicons were analyzed using cysteamine-functionalized gold nanoparticles under fully optimized conditions. (**A**) Representative visual color changes. (**B**) Color value of the samples in (**A**), extracted as the mean A value based on the CIELAB color space (green-red axis). Data are presented as mean ± s.e.m (*n* = 6). At least two independent experiments were performed with similar results. A two-tailed Student’s *t*-test was used to determine statistical significance between the tested samples and the negative control (** *p* ≤ 0.01, *** *p* ≤ 0.001, n.s: non-significant). (**C**) Coefficient of variation calculated from data in (**B**). (**D**) Linear regression analysis of the color value.

As observed in Figure 6A, at high concentrations ranging from 10^8^ to 10^6^ copies of template/reaction, the solution maintains its characteristic vibrant red/pink color. This indicates a dense distribution of amplicons that establishes strong electrostatic interactions, thereby effectively stabilizing the gold nanoparticles against salt-induced aggregation. As the concentration decreases from 10^5^ to 10^2^ copies of template/reaction, the solution color progressively shifts from red to light pink, purple, and ultimately to purple-gray from 10^1^ copies of template and the negative control (Neg) due to electrolyte-driven nanoparticle precipitation.

Regarding the quantitative color value, the bar chart in Figure 6B shows a steady decline in color value as the plasmid concentration decreases. Statistical analysis indicates that from 10^8^ to 10^2^ copies of template/reaction, the color values exhibit statistically significant differences compared to the negative control. At 10^1^ copies of template/reaction, the value shows no statistically significant difference from the Neg control. Therefore, based on both visual inspection and color value data, 10^2^ copies/reaction is determined as the lowest concentration threshold yielding a positive signal distinguishable from the negative control. To confirm the repeatability of the colorimetric assay, we calculated the coefficient of variation (CV%) of the color values for the standard samples (Figure 6C). The CV% values were less than 5%, indicating high assay stability. A linear regression equation based on color value was established, enabling the quantification of TMV cDNA copy numbers in field samples (Figure 6D).

**Figure 7:**
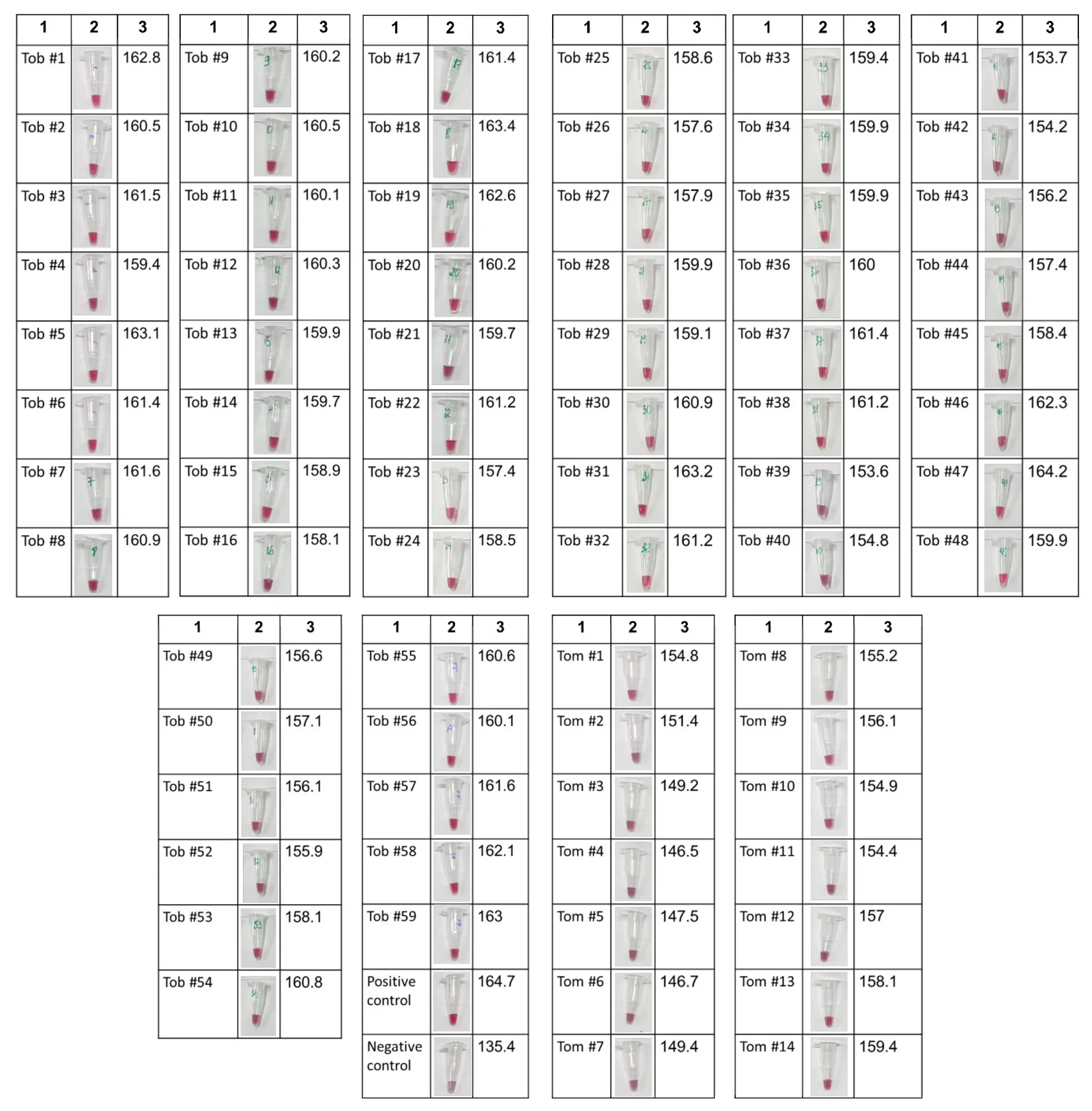
Qualitative results of field samples. A total of 59 tobacco and 14 tomato leaf samples were collected in Cao Bang province, Vietnam, and their total RNA was extracted. For the one-step RT-PCR, 200 ng of RNA was used per 10 µL reaction. DNA template containing 10^8^ copies of the gene encoding the viral coat protein was used as a positive control, while water was used as the negative control. Then 1 µL of RT-PCR products was analyzed using an AuNPs-based colorimetric assay. Columns (1), (2), (3) in the tables represent the sample names (Tob means tobacco, Tom means tomato), representative image results of the colorimetric assay and the color values extracted based on the CIELAB color space (green-red axis), respectively. Color values were presented as mean ± s.e.m (*n* = 4). At least two independent experiments were performed with similar results.

Using the optimized conditions for the colorimetric assay and the linear equation for TMV cDNA quantification, we applied the one-step RT-PCR-coupled gold nanoparticle colorimetric assay to screen 59 tobacco and 14 tomato leaf samples (Figure 7). The color values of all test samples exceeded 144.6, the threshold for distinguishing positive from negative samples (Figure 6B,C). This confirmed that all 73 field samples were positive for TMV. The results were consistent with the agarose gel electrophoresis analysis (Figure S3). However, the uniform positive results across all test samples raised concerns regarding the specificity of the employed primer pair as well as the potential effect of plant matrix components on the colorimetric assay. To verify the specificity of the primer pair—despite being previously published [17]—one-step RT-PCR was performed on a passion fruit leaf sample—a non-host of TMV—infected with East Asian Passiflora virus AO strain (EAPV-AO) and commercial powder of a tomato pericarp sample infected with Tomato spotted wilt virus (TSWV) (Figure S4). Agarose gel electrophoresis revealed no RT-PCR products in the passion fruit and tomato samples when using the TMV-specific primers, and the results were consistent with the colorimetric assay, thereby confirming that our primer pair did not produce non-specific amplicons that could cause false positive results and that plant matrix components did not interfere with the colorimetric results. To further verify the effect of the plant matrix components on the colorimetric assay, we supplemented the RT-PCR reactions with 200 ng of total RNA extracted from a passion fruit leaf sample, which had been confirmed negative for TMV via agarose gel electrophoresis. These reactions contained various copy numbers of the plasmid harboring the TMV coat protein gene as a template (Figure S4C). The color values were not significantly different between the RT-PCR reactions in the absence or presence of the supplementary plant RNA background, further confirming that the plant matrix components did not significantly interfere with the colorimetric assay. Therefore, the linear quantitative regression equation constructed using nuclease-free water background (Figure 6D) can be fully applied to the viral quantification of field samples (Figure S5). The quantification results showed a consistent trend with the band intensity on the agarose gel (Figure S3), as clearly observed in tomato samples number 4, 5, 6, and 7, which exhibited low levels of TMV infection. However, there was an approximately 2-fold difference in the quantified value of the positive sample, with 10^7.7^ copies calculated from the regression equation compared to the 10^8^ copies actually used, indicating that further optimization may be required to improve the viral load quantification accuracy.

### 3.6. Stability of Digital Colorimetric Analysis Under Varying Lighting Conditions

To evaluate the reliability, reproducibility, and practical adaptability of the developed digital color extraction system, we investigated the stability of color value extraction under different lighting conditions. The colorimetric assay was performed on ten field samples, including five tobacco samples and five tomato samples, alongside a positive control (10^7^ copies of the TMV plasmid) and a negative control (nuclease-free water). Image capture was carried out at a fixed shooting angle of approximately 90° and a distance of 40 cm away from the surface, under four distinct light intensities, including 260 Lux, 420 Lux, 600 Lux—the light intensity routinely used throughout this study and 690 Lux. To ensure statistical rigor, the assays were conducted across two independent RT-PCR trials, with each run analyzed in triplicate (*n* = 6 per sample per lighting condition).

The quantitative color values extracted via our open-source GitHub algorithm (ColorChoose_v1.0.0) are summarized in Table 1. The system demonstrated exceptional repeatability, with the intra-group coefficients of variation (CV%_intra group) consistently remaining below 2% across all tested light intensities. Furthermore, the inter-group coefficients of variation (CV%_inter group) across the different lighting environments were strictly less than 3%, indicating that the digital color analysis is highly robust and immune to typical variations in ambient lighting.

Importantly, the color value thresholds (144.6) established for distinguishing positive and negative samples (Figure 6) remained fully applicable and highly accurate under all tested light intensities. However, we noted that under low-light conditions (260 Lux), samples with mild TMV infections, such as tomato samples Tom#4 and Tom#5, require careful evaluation. These results confirm that the developed digital quantitative color analysis is stable, highly adaptable, and well-suited for field-deployable diagnostics.

## 4. Discussion

In this study, we successfully optimized a procedure for the visual detection of an RNA virus, which reduces diagnostic time and the need for expensive devices. The two steps, including cDNA synthesis and the amplification reaction, were combined in a single tube, significantly saving time. The resulting RT-PCR products were subsequently analyzed using an AuNPs-based colorimetric assay without the need for optical devices for agarose gel analysis. Here, our AuNPs were functionalized with cysteamine to enhance the color value difference between positive and negative samples. The optical enhancement can be explained by the ligand exchange process, where cysteamine partially replaces citrate molecules on the AuNP surface via the formation of strong Au–S bonds [19]. The terminal amine groups of cysteamine reduce the net negative surface charge of the AuNPs and introduce positively charged sites, facilitating stronger interactions with negatively charged dsDNA molecules. This surface modification significantly enhances the adsorption efficiency of dsDNA onto the AuNP surface, enabling the formation of a stable colloidal state even in the presence of high salt concentrations (Figure 1 and Figure 2).

In addition, using thiolated primers enhanced the colorimetric contrast between positive and negative controls (Figure 3). This is due to the strong affinity of the thiol group in dsDNA amplicons to the gold surface via Au-S bonding. Interestingly, the thiolated forward primer alone showed a significantly higher color value difference compared to non-thiolated primers. Furthermore, a distinct behavior was observed depending on whether the thiolated forward or the thiolated reverse primer was used (Figure 3C). This may occur due to differences in the DNA sequence (A, T, C, G) at 5′ and 3′ends proximal to the thiolated region, resulting in different binding affinities of the DNA amplicons to the AuNP surface [20,21]. Considering the additional synthesis cost associated with dual-thiol modification, employing a single thiolated forward primer, which has demonstrated clear color discrimination between positive and negative samples, represents a cost-effective alternative for practical field applications. In our procedure, we also found that incubating DNA with AuNPs at RT or 37 °C for just 5 min can achieve the best visual discrimination between positive and negative samples (Figure 5), enabling viral diagnosis with cheap equipment and a short turnaround time.

Notably, the sensitivity of our procedure surpassed that of the traditional agarose gel analysis, achieving a limit of detection (LOD) of 10^2^ copies of TMV cDNA compared with 10^3^ copies for the gel-based method (Figure 6 and Figure S2). The superior sensitivity of the colorimetric assay stems from the unique optical properties of gold nanoparticles (AuNPs). AuNPs possess exceptionally high extinction coefficients, up to a million times greater than those of organic dyes used in agarose gel electrophoresis, allowing them to intensely absorb and scatter light even at nanomolar concentrations [22,23]. Furthermore, while agarose gel electrophoresis is a separation-based method that can cause diffusion and smearing of DNA molecules (resulting in signal loss), the colorimetric assay is performed in-tube. This in-tube approach allows the reaction to maintain equilibrium without diluting the signal. Our colorimetric assay also showed good specificity with no cross-amplification of other plant viruses such as EAPV-AO and TSWV. However, in the present study, we were unable to experimentally examine the specificity of our assay against other viruses belonging to the same family as TMV. This was because obtaining TMV-free samples that were concurrently infected with other tobamoviruses proved challenging, due to the exceptionally wide host range and highly contagious nature of TMV. Nevertheless, a sequence alignment of our primers against the genomes of several prevalent tobamoviruses via NCBI BLAST (https://blast.ncbi.nlm.nih.gov/Blast.cgi) accessed on 7 July 2026, including Tobacco mild green mosaic virus (TMGMV), Tomato mosaic virus (ToMV), Tomato Mottle Mosaic Virus (ToMMV), Cucumber green mottle mosaic virus (CGMMV), Tomato brown rugose fruit virus (ToBRFV), and Pepper mild mottle virus (PMMoV), demonstrated that our TMV-specific primer pair is highly unlikely to yield non-specific amplicons under our optimized RT-PCR conditions. Regarding repeatability, our assay demonstrated excellent reproducibility, characterized by low coefficient of variation values (Figure 6C) and robust stability in the presence of interfering factors such as plant RNA background or variations in light intensities (Figure S4 and Table 1). However, the viral load quantification via the regression equation may need further optimization to improve the accuracy. This could be achieved by constructing a regression equation with a higher resolution by incorporating intermediate plasmid standard concentrations, specifically supplementing points at 10^7.5^, 10^6.5^, 10^5.5^, 10^4.5^, 10^3.5^, 10^2.5^, 10^1.5^ copies or by developing alternative mathematical models to better capture the relationship between color values and viral loads.

This study also screened a substantial number of field samples, including 59 tobacco and 14 tomato leaf samples collected from a Northern province of Vietnam (Figure 7). Remarkably, all tested samples were positive for TMV using both the colorimetric assay and agarose gel analysis. This high prevalence underscores the extensive spread of TMV, which poses a severe threat to various vital crops both in Vietnam and globally [5,24,25,26]. Our research, therefore, is highly significant and lays a foundation for the further development of cheap and rapid diagnostic tests to detect plant viruses.

## 5. Conclusions

Based on the optimized conditions established throughout this study, we developed a workflow for TMV detection as shown below:

1. Preparation
  - RT-PCR: Total RNA is extracted from leaf samples and amplified via a 10 µL RT-PCR reaction using 200 ng of template RNA.
  - Functionalized Nanoparticles: Stock AuNP solution is thoroughly vortexed, diluted 2.5-fold (*v*/*v*) with deionized water, and mixed with 0.01 mM cysteamine at a 4:1.75 (*v*/*v*) ratio. The mixture (Au@Cys) is incubated at room temperature for at least 1 h.
2. Colorimetric Assay
  - Reaction: 1 µL of the RT-PCR product is diluted with 9 µL of nuclease-free water. Then, 10 µL of this diluted product is mixed with 10 µL of the prepared Au@Cys solution and incubated for 5 min at room temperature.
  - Salt Induction and Imaging: 4 µL of 5 M NaCl is added to trigger the aggregation. Color shifts are recorded 10 min post-NaCl addition under controlled lighting, approximately 600 Lux, with the camera positioned 40 cm away at a 90-degree angle to the surface.
3. Digital color analysis:
  - Image Processing: Images are uploaded to the open-source ColorChooser software (ColorChooser_v1.0.0) (https://github.com/Thaonguyennnee/ColorChooser) accessed on 22 May 2026. Identical Regions of Interest (ROIs) are selected from the center of each liquid solution.
  - Qualitative and quantitative analysis of viral infection: The qualitative results can be observed with the naked eye. However, for greater accuracy, sample color values were extracted as the mean A value of the selected liquid region based on CIELAB color space (green-red axis). The samples with color values ≥ 144.6 were determined as positive for TMV, whereas those below this threshold were classified as negative. For viral load quantification, the color values can then be applied to the linear equation of y = 3.5078x + 137.42. It should be noted that for more accurate results, the standard curve may need to be re-constructed using our method under specific local parameters.

This simple framework successfully detects TMV infection at both qualitative and quantitative levels.

## Figures and Tables

**Table 1:** Color values extracted from TMV diagnostic colorimetric assay under varying light intensities (*n* = 6). Coefficient of variation values were calculated for intra-group and inter-group.

Light Intensity (Lux)		Tob#1	Tob#2	Tob#3	Tob#4	Tob#5	Tom#1	Tom#2	Tom#3	Tom#4	Tom#5	Positive	Negative
260	Color value mean	159.9	155.4	158.3	154.6	158.9	151.2	149.1	148.4	144.6	144.3	157.5	136.8
	SD	1.0	0.8	0.7	0.7	1.0	1.0	1.7	0.9	0.8	1.4	1.4	0.7
	CV%_intra group	0.6	0.5	0.5	0.5	0.6	0.7	1.1	0.6	0.5	1.0	0.9	0.5
420	Color value mean	164.6	160.2	164.4	160.4	164.9	155.0	150.9	150.1	146.6	145.6	162.6	135.5
	SD	0.7	0.9	0.6	1.0	1.0	1.5	1.1	1.3	1.1	1.2	0.3	1.0
	CV%_intra group	0.5	0.6	0.4	0.6	0.6	1.0	0.7	0.9	0.7	0.8	0.2	0.8
600	Color value mean	162.8	160.5	161.5	159.4	163.1	154.8	151.4	149.2	146.5	147.5	159.8	133.7
	SD	1.6	1.5	0.7	1.5	1.3	1.2	0.8	1.2	0.9	1.4	1.5	1.0
	CV%_intra group	1.0	0.9	0.4	0.9	0.8	0.7	0.6	0.8	0.6	1.0	1.0	0.7
690	Color value mean	167.0	163.3	166.6	162.6	167.8	155.2	152.0	150.7	147.0	147.5	165.1	136.9
	SD	0.9	1.3	1.5	1.2	2.1	3.7	3.0	2.8	1.9	2.5	3.2	2.8
	CV%_intra group	0.6	0.8	0.9	0.7	1.3	2.4	2.0	1.8	1.3	1.7	1.9	2.0
	CV%_inter group	1.8	2.0	2.2	2.1	2.3	1.2	0.8	0.7	0.7	1.1	2.1	1.1

## Data Availability

The data that support the findings of this study are available on request from the corresponding author.

## Supplementary Materials

The following supporting information can be downloaded at: https://www.mdpi.com/article/10.3390/mps9040111/s1, Figure S1: Morphological characterization of gold nanoparticles at different functionalization stages. Representative TEM images of (A) bare citrate-stabilized AuNPs, (B) cysteamine-functionalized AuNPs (Au@Cys), and (C) Au@Cys following incubation with dsDNA PCR products (Au@Cys + DNA). (D) Absorption spectra of bare citrate-stabilized AuNPs and Au@Cys before and after reacting with RT-PCR products and salt induction. (E) Zeta potential of gold nanoparticles before and after cysteamine functionalization; Figure S2: One-step RT-PCR results of standard samples using agarose gel electrophoresis analysis. Plasmid templates containing 10^8^ to 10^6^ copies of the gene encoding viral coat protein were used for one-step RT-PCR. Next, 1 µL of RT-PCR products was analyzed using 2% agarose gel. At least two-independent experiments were done with similar results; Figure S3: One-step RT-PCR results of field samples using agarose gel electrophoresis analysis. (A–D) Results of 59 Tobacco leaf samples. (E) Results of 14 Tomato leaf samples. 400 ng total RNA extracted from leaf samples were used for one-step RT-PCR. Positive controls used plasmid templates containing 10^8^ and 10^6^ copies of the gene encoding viral coat protein. Water was used as negative control. 1 µL of RT-PCR products from Tobacco leaf samples and 5 µL of RT-PCR products from Tomato leaf samples were used for agarose gel electrophoresis analysis; Figure S4: One-step RT-PCR combining gold nanoparticle-based colorimetric assay specifically detect Tobacco mosaic virus infected in plant tissues. (A) One-step RT-PCR results. 400 ng total RNA extracted from each samples were used for one-step RT-PCR. Next, 1 µL of RT-PCR products was analyzed by 2% agarose gel electrophoresis. Lane 1: Tomato pericarp sample infected with *Tomato spotted wilt virus* (TSWV), using a TSWV-specific primer pair to amplify 684 bp products. Lane 2: Tomato pericarp sample infected with TSWV, using the TMV-specific primer pair. Lane 3: Passion fruit leaf sample infected with *East Asian Passiflora virus* AO strain (EAPV-AO) virus, using an EAPV-AO-specific primer pair to amplify 524 bp products. Lane 4: Passion fruit leaf sample infected with EAPV-AO virus, using the TMV-specific primer pair. Lane 5: Tobacco leaf sample infected with TMV, using the TMV-specific primer pair. Lane 6: Negative control (distilled water as template). (B) AuNPs-based colorimetric results of the RT-PCR products from lane 2, lane 4 and lane 5. (C) Colorimetric results of RT-PCR products using 10^7^, 10^5^ and 10^3^ plasmid copies as template, in the absence or presence of supplementary passion fruit leaf RNA. Nuclease-free water was utilized as the template for the negative control. The experiment was conducted in three independent RT-PCR trials, with the products of each run analyzed in duplicate (*n* = 6). A two-tailed Student’s *t*-test was used to determine statistical significance between samples with and without supplementary plant RNA (n.s.: non-significant); Figure S5: Quantification results of 73 field samples in Figure 7. Color values extracted based on CIELAB color space were applied to linear regression equation in Figure 6 to calculate the TMV cDNA load. Column (1) and (2) in the tables represent the sample names (Tob means tobacco, Tom means tomato) and the quantification results, respectively; Table S1: Sequences of specific primers for *East Asian Passiflora virus* AO strain (EAPV-AO) and *Tomato spotted wilt virus* (TSWV).
