# Supplementary material for: A One-Step RT-PCR-Coupled Cysteamine-Functionalized Gold Nanoparticle Assay for Colorimetric Detection of Tobacco Mosaic Virus
Source: Methods Protoc. 2026 Jul 27;9(4):111. doi: 10.3390/mps9040111 (PMC13515312; doi:10.3390/mps9040111)
Supplement: Supplementary file 1 [file mps-09-00111-s001.zip › mps-4417019-supplementary.pdf]

A One-Step RT-PCR-Coupled Cysteamine-Functionalized Gold Nanoparticle Assay for Colorimetric Detection of Tobacco Mosaic Virus

Supplemetary Documents

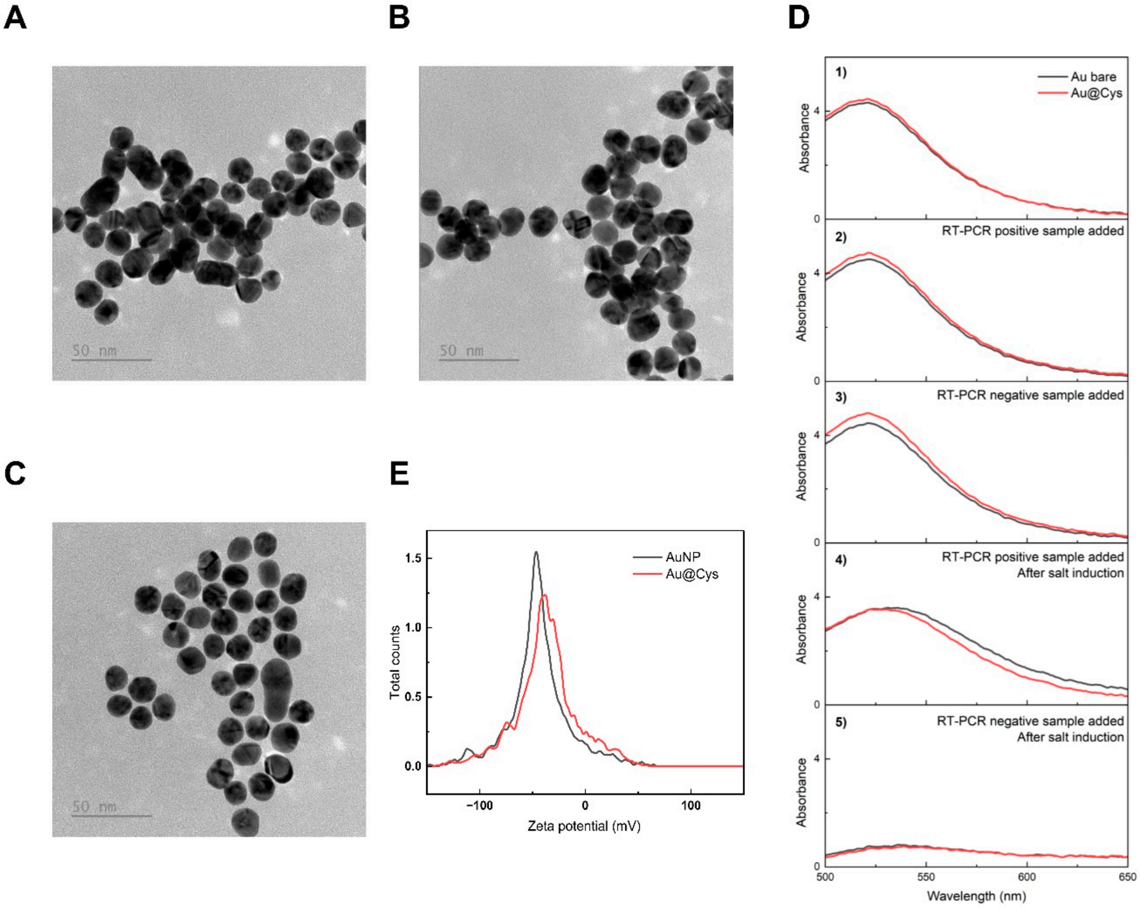

Supplementary Figure S1. Morphological characterization of gold nanoparticles at different functionalization stages. Representative TEM images of (A) bare citrate-stabilized AuNPs, (B) cysteamine-functionalized AuNPs (Au@Cys), and (C) Au@Cys following incubation with dsDNA PCR products (Au@Cys+DNA). (D) Absorption spectra of bare citrate-stabilized AuNPs and Au@Cys before and after reacting with RT-PCR products and salt induction. (E) Zeta potential of gold nanoparticles before and after cysteamine functionalization.

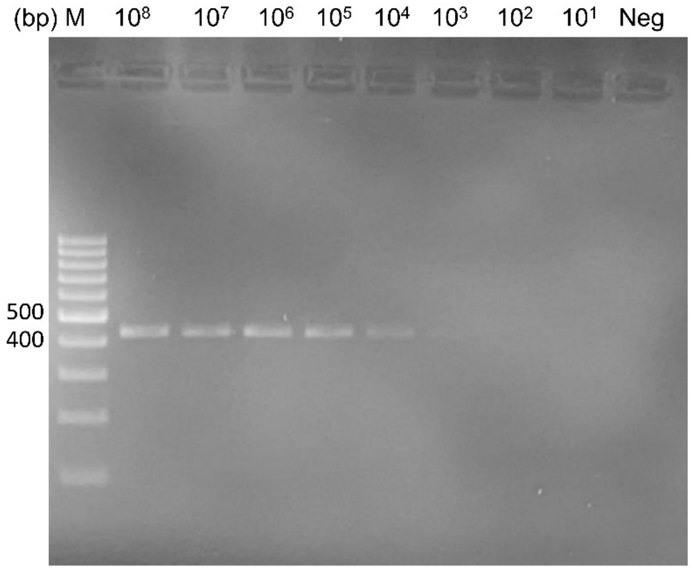

Supplementary Figure S2. One-step RT-PCR results of standard samples using agarose gel electrophoresis analysis. Plasmid templates containing 10^8 to 10^6 copies of the gene encoding viral coat protein were used for one-step RT-PCR. Next, 1 μL of RT-PCR products was analyzed using 2% agarose gel. At least two-independent experiments were done with similar results.

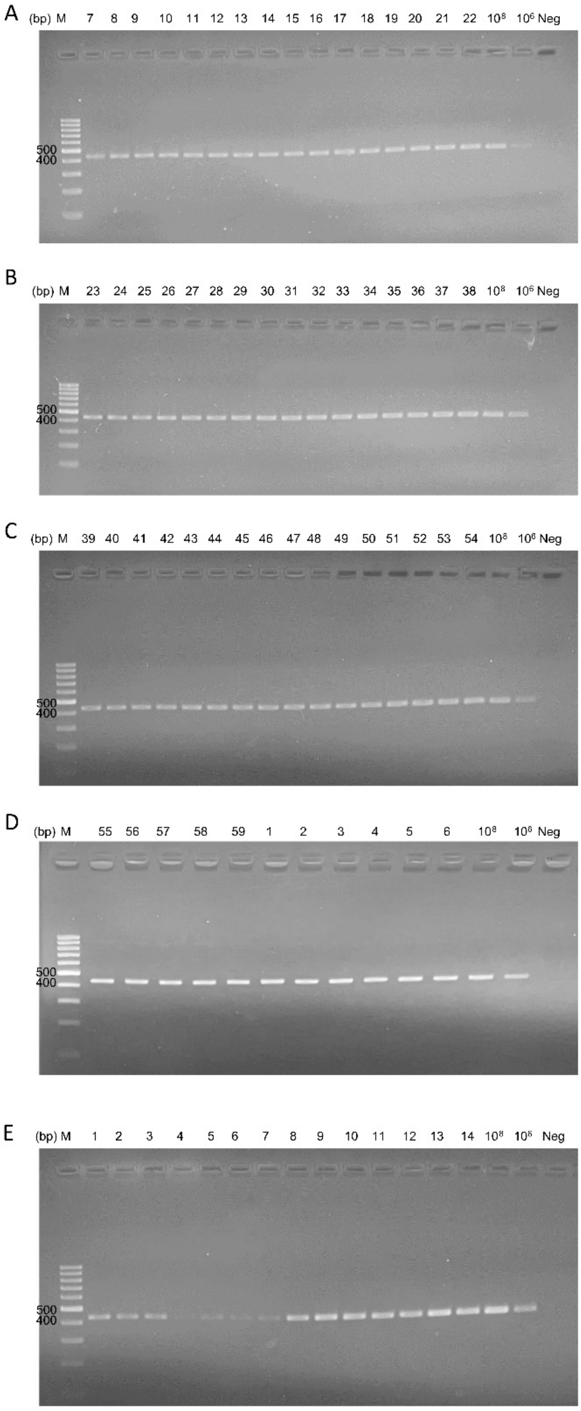

Supplementary Figure S3. One-step RT-PCR results of field samples using agarose gel electrophoresis analysis. (A-D) Results of 59 Tobacco leaf samples. (E) Results of 14 Tomato leaf samples. 400 ng total RNA extracted from leaf samples were used for one-step RT-PCR. Positive controls used plasmid templates containing 10^8 and 10^6 copies of the gene encoding viral coat protein. Water was used as negative control. 1 μL of RT-PCR products from Tobacco leaf samples and 5 μL of RT-PCR products from Tomato leaf samples were used for agarose gel electrophoresis analysis.

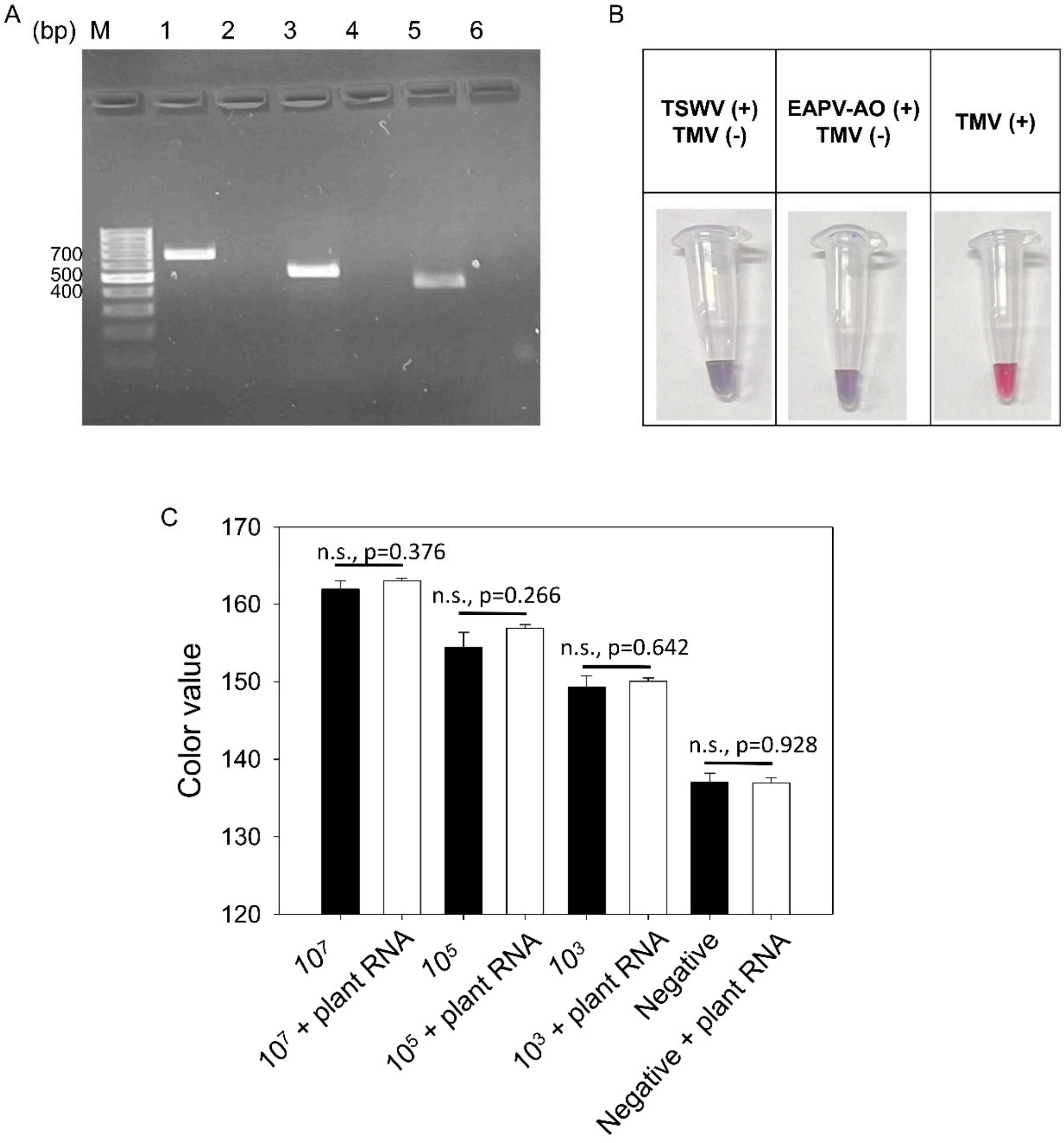

Supplementary Figure S4. One-step RT-PCR combining gold nanoparticle-based colorimetric assay specifically detect Tobacco mosaic virus infected in plant tissues. (A) One-step RT-PCR results. 400 ng total RNA extracted from each samples were used for one-step RT-PCR. Next, 1 μL of RT-PCR products was analyzed by 2% agarose gel electrophoresis. Lane 1: Tomato pericarp sample infected with *Tomato spotted wilt virus* (TSWV), using a TSWV-specific primer pair to amplify 684 bp products. Lane 2: Tomato pericarp sample infected with TSWV, using the TMV-specific primer pair. Lane 3: Passion fruit leaf sample infected with *East Asian Passiflora virus* AO strain (EAPV-AO) virus, using an EAPV-AO-specific primer pair to amplify 524 bp products. Lane 4: Passion fruit leaf sample infected with EAPV-AO virus, using the TMV-specific primer pair. Lane 5: Tobacco leaf sample infected with TMV, using the TMV-specific primer pair. Lane 6: Negative control (distilled water as template). (B) AuNPs-based colorimetric results of the RT-PCR products from lane 2, lane 4 and lane 5. (C) Colorimetric results of RT-PCR products using 10^7, 10^5 and 10^3 plasmid copies

as template, in the absence or presence of supplementary passion fruit leaf RNA. Nuclease-free water was utilized as the template for the negative control. The experiment was conducted in three independent RT-PCR trials, with the products of each run analyzed in duplicate (n = 6). A two-tailed Student's t-test was used to determine statistical significance between samples with and without supplementary plant RNA (n.s.: non-significant).

1	2
Tob #1	$10^{7.2}$
Tob #2	$10^{6.6}$
Tob #3	$10^{6.9}$
Tob #4	$10^{6.3}$
Tob #5	$10^{7.3}$
Tob #6	$10^{6.8}$
Tob #7	$10^{6.9}$
Tob #8	$10^{6.7}$

1	2
Tob #9	$10^{6.5}$
Tob #10	$10^{6.6}$
Tob #11	$10^{6.5}$
Tob #12	$10^{6.5}$
Tob #13	$10^{6.4}$
Tob #14	$10^{6.3}$
Tob #15	$10^{6.1}$
Tob #16	10^{6}

1	2
Tob #17	$10^{6.8}$
Tob #18	$10^{7.4}$
Tob #19	$10^{7.2}$
Tob #20	$10^{6.5}$
Tob #21	$10^{6.3}$
Tob #22	$10^{6.8}$
Tob #23	$10^{5.7}$
Tob #24	10^{6}

1	2
Tob #25	10^{6}
Tob #26	$10^{5.8}$
Tob #27	$10^{5.8}$
Tob #28	$10^{6.4}$
Tob #29	$10^{6.2}$
Tob #30	$10^{6.9}$
Tob #31	$10^{7.4}$
Tob #32	$10^{6.8}$

1	2
Tob #33	$10^{6.3}$
Tob #34	$10^{6.4}$
Tob #35	$10^{6.4}$
Tob #36	$10^{6.4}$
Tob #37	$10^{6.8}$
Tob #38	$10^{6.8}$
Tob #39	$10^{4.6}$
Tob #40	$10^{4.9}$

1	2
Tob #41	$10^{4.6}$
Tob #42	$10^{4.8}$
Tob #43	$10^{5.4}$
Tob #44	$10^{5.7}$
Tob #45	10^{6}
Tob #46	10^{7}
Tob #47	$10^{7.6}$
Tob #48	$10^{6.4}$

1	2
Tob #49	$10^{5.5}$
Tob #50	$10^{5.6}$
Tob #51	$10^{5.3}$
Tob #52	$10^{5.3}$
Tob #53	$10^{5.9}$
Tob #54	$10^{6.7}$

1	2
Tob #55	$10^{6.6}$
Tob #56	$10^{6.5}$
Tob #57	$10^{6.9}$
Tob #58	10^{7}
Tob #59	$10^{7.3}$
Positive control (10^{8} copies)	$10^{7.7}$
Negative control	$10^{-0.85}$

1	2
Tom #1	$10^{4.9}$
Tom #2	$10^{3.9}$
Tom #3	$10^{3.4}$
Tom #4	$10^{2.6}$
Tom #5	$10^{2.9}$
Tom #6	$10^{2.6}$
Tom #7	$10^{3.4}$

1	2
Tom #8	$10^{5.1}$
Tom #9	$10^{5.3}$
Tom #10	10^{5}
Tom #11	$10^{4.8}$
Tom #12	$10^{5.6}$
Tom #13	$10^{5.9}$
Tom #14	$10^{6.3}$

Suppementary Figure S5. Quantification results of 73 field samples in Figure 7. Color values extracted based on CIELAB color space were applied to linear regression equation in Figure 6 to calculate the TMV cDNA load. Column (1) and (2) in the tables represent the sample names (Tob means tobacco, Tom means tomato) and the quantification results, respectively.

Supplemetary Table S1. Sequences of specific primers for *East Asian Passiflora virus* AO strain (EAPV-AO) and *Tomato spotted wilt virus* (TSWV).

Primers	Sequence (5’-3-)	Product size
EAPV-AO-F	CCCATGGTATTCAGCAGTCCAAAGAG	524 bp
EAPV-AO-R	GGGAATTCATACCCAAAAGGGTGT	
TSWV-F	TTTAGCATTAGGATTGCTGG	684 bp
TSWV-R	ATCATGTCTAAGGTTAAGCTC	
